# Die Lehre von der Reflex-Function für Physiologen und Aerzte

**Published:** 1843-01-01

**Authors:** 


### Die Lehre von der Reflex-Function für Physiologen und Aerzte; Dargestellt und beurtheilt von *Johann Wilhelm Arnold.* The Theory of the Reflex Function, represented and examined by John Wm. Arnold. *Heidelberg*, 1842.

The object of this book seems to be neither more nor less, than to prove, that the Theory of the Reflex-Function, which from its novelty has made of late years so great a noise in the scientific world, is no novelty at all; and that the facts upon which the modern abettors of this theory have erected it, were as well known to several of the old physiologists, as to those of the present day. The author indeed goes so far as to state, that, if M. Hall and J. Müller had studied the history of their science with sufficient care, we should have been spared much of the trouble and fuss occasioned by the regeneration of a doctrine "as old as the hills."

In his first Chapter the author presents us with a very succinct view of the *Reflex Theory of Hall and Müller.* Hall distinguishes four species of muscular motion. The *first* is the *Voluntary:* the will, taking its origin in the brain, and free in its action, extends its influence along the spinal chord and the moving nerves in a direct line to the voluntary muscles. The *second* is the *Respiratory*, which has its origin in the medulla oblongata. The *third* species of motion is the *Involuntary;* this depends on irritability, and requires the immediate action of an irritant on the muscular fibres. These three species of muscular motion have been known for a long time, and were the only species known, according to Marshall Hall. According to this physiologist, however, there is a *fourth* species which still continues, after the voluntary and respiratory motions have ceased by the removal of the brain and medulla oblongata; this species of motion is connected with the spinal marrow, and disappears as soon as this organ is taken away, though irritability still continues. In this species of muscular motion the acting influence exists not in a central part of the nervous system, but at a distance from it. It is neither voluntary in its action, nor direct in its course; it is, on the contrary, excited by peculiar stimuli, which, however, do not come into immediate contact with the muscular fibre, but with certain membranous parts, from whence the impression is conducted to the spinal marrow, is thence reflected, and either again reaches the part on which the impression took place, or some other part remote from it, where muscular contractions now take place. This is M. Hall's reflex-motion. It exists as an uninterrupted muscular action, as a power which presides over organs, which are not really in a state of motion, and in some, as in the larynx, it presides over, and preserves an opening, whilst in other parts, as in the sphincter muscles, it keeps the organs closed. In the reflex-function the muscles are excited by a stimulus, which is conducted mediately and indirectly in an arched and reflected course along the superficial nerves under the skin or mucous membrane to the spinal marrow, and along the muscular nerves from the spinal marrow. The motion that takes place in this way requires, that the connexion with the spinal marrow be uninjured. M. Hall supported his theory chiefly by experiments on cold-blooded animals, which go to show that, after decapitation, suitable and determinate motions take place in consequence of the action of external stimuli, but not from free impulse, but that these motions cease, as soon as the spinal marrow is removed. M. Hall draws from his experiments with decapitated animals the following conclusions, which are to serve as supports and illustrations of his theory :

1st. Sensation can act for the production of muscular motion only by means of the will.

2nd. In the experiments with the removal of the brain and medulla oblongata, the will is no doubt abolished, but not the moving power.

3rd. In cases where sensation is shut out and the will is abolished, the external impressions which occasion pain, act on a property of the nervous system, which is different from sensation.

The observations according to which frogs and salamanders thrown into a state of rigid spasm by nux vomica or opium, may be divided into three parts, namely, the head, the anterior, and the posterior extremities, without the increased irritability and spasm ceasing, should, according to M. Hall, sufficiently prove, that the phenomena, which he ascribed to the reflex-function, depend neither on sensation, nor on the will, nor on irritability. He considers it evident that the spasmodic phenomena in tetanus are no voluntary motions, that they obey the same laws as those motions which are observed in an animal slightly affected with tetanus, or in parts of such an animal under the influence of stimuli. It is, according to him, equally clear, that phenomena which depend on the excitement of irritability, would not cease, as long as this irritability continues uninjured. M. Hall connects with his reflex-theory a peculiar theory on the structure of the nervous system, namely, of the spinal marrow and its nerves. He assumes, for instance, an excito-motory nervous system, and from his experiments infers the existence: 1st, Of a proper spinal marrow which is physiologically distinct from the chord of the intro-spinal nerves ; 2, of a system of excito-motory nerves, physiologically distinct from that of the sensitive and voluntary nerves : 3, of a nervous power, the excito-motory power, which acts, being thrown in an incident direction, upwards, downwards, and backwards, in reference to the proper spinal marrow, the centre of the excito-motory system.

Thus then the entire spinal marrow in vertebrated animals consists of two parts, the first being the intervertebral chord of sensitive and voluntary nerves, which go to and from the brain, as their middle point; the second has been called the true spinal marrow and is excito-motory. This is the axis of the system of excito-motory nerves, which are generally, but probably not invariably, connected with the former.

The parts of the excito-motory system of M. Hall are : 1. The incident excitory branches : 1. The trifacial,—2. The pneumo-gastric.—3. The posterior spinal nerves.

2. The true medulla oblongata and spinal marrow, the middle point of this system.

3. The reflecting, moving branches, as the trochlearis, abducens oculi, &c. &c.

Among the Germans, J. Müller was the first who advocated this theory. He differs, however, on many points from M. Hall. His views on reflexion are as follows : when sensations which are effected through external stimuli on nerves of sensation, produce motions in other parts, this never takes place through the reciprocal action of the sensitive and motory fibres of a nerve, but by the sensorial excitement acting on the brain and spinal marrow, and from this back on the motory fibres. The phenomenon of general convulsions after local sensations is independent of the N. sympatheticus, and is occasioned by an irritation of the spinal marrow, whereby every local, sensorio-centripetal excitement transplants itself to the entire spinal marrow and brain, and thence necessarily excites all the motory fibres. But in very many cases after local irritation of the nerves, not general but local, convulsive twitches are occasioned, which, however, must always be explained, and accounted for, through the spinal marrow, as the connecting medium between the sensorial and motory fibres. With respect to M. Hall's view, according to which no sensation takes place in the case of motions communicated through the spinal marrow, J. Müller thinks, that the reflected motions which take place on the application of stimuli to the skin after the removal of the brain, contain no proof that cutaneous irritants are still capable of exciting true sensation in the spinal marrow; it is rather the centripetal conducting of the nervous principle which ordinarily takes place in the case of sensations, but which in this case is no longer a sensation, as it is no longer conducted to the brain, the organ of consciousness. During health many reflected motions follow through cutaneous irritants, which do not reach consciousness, as true sensations, but still are capable of making violent impressions on the spinal marrow. But Müller thinks that M. Hall goes too far in assuming that, in health, every motion in consequence of a true sensation is produced by the will, and that all excitements of sensible parts in reflected motions are without sensation; for the reflected motions of sneezing, coughing, and several others follow from real sensation. According to Müller an irritation of a sensorio-spinal nerve in the first instance effects a centripetal action of the nervous principle towards the spinal marrow. If this can reach the sensorium, it is a sensation, attended with consciousness. But if, on account of a division of the spinal marrow, it does not reach the sensorium commune, it still retains its entire power as a centripetal action to the spinal marrow. In both cases, a centripetal action of a sensorial nerve may produce a reflex motion. In the former case the centripetal action would be at the same time a sensation, in the latter not, but it suffices for a reflex-motion. We possess, according to Müller, no certain facts to prove that the spinal marrow is endowed with sensation independently of the brain and medulla oblongata. Reflex-motions after cutaneous irritations in decapitated animals cannot, according to him, be reckoned among these, and if decapitated frogs evince, on the application of a cutaneous irritant, any thing determinate or suitable in their reaction, this phenomenon only occurs when the division through the spinal marrow occurs at its commencement.

With respect to the manner in which the spinal marrow is the connecting medium between a sensorial and motory motion of the nervous principle, Müller assumes that it is the easiest way of oscillation or vibration from the posterior root of a nerve or of its individual primitive fibres to its anterior root, or to the anterior roots of several adjacent nerves. The principle of the nerves in these vibrations or oscillations takes the readiest way, in order to act from sensitive nerves on motory fibres through the spinal marrow. Another very usual track of conduction from sensitive nerves to motory nerves, through the medium of the spinal marrow and medulla oblongata, is that of excitation of the mucous system, and of the secondary affection of the respiratory muscles in vomiting, tenesmus, parturition, dysuria, coughing, sneezing, &c. Müller thinks, that there must be previously formed in the medulla oblongata and spinal marrow, between the sensitive nerves of the mucous system and the motory nerves of respiration an easier conduction, whilst, on the contrary, the spinal nerves going to the extremities are excluded from this harmony.

Müller distinguishes two principal groups of reflex-motions:—

A. Reflex-motions of the animal system, wherein he reckons the reflex-motions of the muscles supplied with cerebral and spinal nerves; the centripetal excitation may be produced in the animal or organic nerves, in the external skin, or in the intestinal canal.

B. Reflex-motions of the organic system. To these belong, according to him, the reflex-motions of the involuntary moving muscles; the centripetal excitation may now be transferred, first to the brain and spinal marrow from cerebral and spinal nerves, or may have proceeded from organs which are provided from the organic nervous system.

The laws of reflexion, which were established in the case of the cerebro-spinal nerves, hold good, according to Müller, likewise with respect to the sympathetic nerves; that is, violent impressions of sensation in parts supplied by the sympathetic may, when transferred to the spinal marrow, produce motions in parts supplied by cerebro-spinal nerves. Reflexion from impressions of sensation in parts provided by the sympathetic to the spinal marrow and brain, and from thence to the motory action of the sympathetic, takes place also, but in a less degree, than in the cerebro-spinal nerves. The reflexion also of effects or actions, which proceed from the cerebro-spinal nerves, are transferred to the spinal marrow, and from thence to the sympathetic nervous system, is, according to Müller, a tolerably frequent phenomenon. A reflecting power in the ganglia, in the case of sympathetic sensations, according to the same author, is not proved, and several facts are opposed to it. He even assumes it as probable, that the brain and spinal marrow are the connecting medium, when secretions follow in distant parts after sensations through reflexion.

In order clearly to understand Müller's views concerning reflex-motion, it is necessary to consider how, according to him, the voluntary motions take place; as also his physiology of the spinal chord. The spinal nerves are, according to him, capable of voluntary determination, in this way; the fibres of the spinal nerves ascend upwards in the spinal marrow, and are exposed to the influence of the will in the medulla oblongata, the source of all voluntary motion. On the other hand, the activity of the motory cerebral nerves receives the impulse to voluntary motions from the medulla oblongata. We may suppose that, in this part of the brain, the fibres of all the motory cerebral nerves and of the spinal nerves are unfolded. The will puts these fibrous origins into action, like the keys of a harpsichord. To the voluntary motion there is wanting only the excitement of a vibration or an oscillation in the origins of a certain quantity of the fibres of the medulla oblongata. All the rest is mere mechanism. In another place, Müller designates the brain as the source of sensations and voluntary motions, when he says: the brain receives the impressions of all the sensitive fibres of the entire system, becomes conscious of them, and knows the place of the sensation according to the affection of the various primitive fibres; the brain again excites the motory powers of all the motory primitive fibres and of the spinal marrow in the case of voluntary motion. So various is the power, and still so similar is the action of the brain in exciting a certain part under the immense number of primitive fibres to the playing of a many-stringed instrument, the strings of which sound as the keys are touched. The mind is the performer, the primitive fibres of all the nerves, which are diffused over the brain, are the strings, and the commencements of these are the keys.

In man and the higher animals the spinal marrow, according to Müller, bears exactly the same relation to the brain, that all the cerebral nerves bear to it, and the spinal marrow is to be considered as a common stock of all the nerves of the trunk, though it still possesses peculiar powers before the nervous stems. The spinal marrow represents not only all the nerves of the trunk in general in the brain, but also the individual primitive fibres of the nerves of the trunk. The primitive fibres of the nervous stocks which enter into the spinal marrow, are not bound together, according to him, in the spinal marrow, but proceed in a direction parallel to each other, as in the stock of a nerve, to the brain, in order that thus isolated and separate, they may impart to this organ local sensations, and that in this isolated state also they may receive excitation for motion. For, says Müller, if the primitive fibres of the nerves were bound together in the spinal marrow, a local sensation in the trunk would be just as little possible, as a separate and isolated contraction of separate muscles on the trunk. The seat of the sensations is neither in the nerves, which bring to the brain the oscillations or vibrations of the nervous principle necessary thereto, nor in the spinal marrow, which conducts these effects or actions, like nerves, to the sensorium commune; this takes place only through the action of the fibres of the nerves and of the spinal marrow on the sensorium commune. But the spinal marrow serves not only as a conductor of the fibres of the nerves to the brain, but appears also as part of the central organ. As such, it possesses the power of reflecting the sensorial irritations of its sensitive nerves to the motor nerves. It possesses also the property, whereby motions follow a sensation, without both kinds of nerves communicating by their primitive fibres, a property possessed by no nerve that is separated from the central parts. The spinal marrow is capable of reflexion from nerves of sensation to nerves of motion without itself possessing sensation.

### Views of Several Physiologists of Modern Times on the Reflex Theory.

Volkmann,* though he values highly the very important discoveries of

---

* See Medico-Chirurgical Review, No. for July 1838, where an analysis of Volkmann's Paper may be found.—*Rev.*

M. Hall, still considers many of his conclusions not well grounded. He first objects to the assertion that decapitated animals continue at rest in the place where they are put, and continue therein unchanged to the extinction of the very last spark of life. He has proved also by experiments that a longitudinal division of the spinal marrow does not prevent the extension of the reflex-motions over all the muscles of both halves of the body, so long as any part of the proper spinal marrow continues connected in the middle line. He also infers from his experiments that the reflex-motions have the character of aiming at some end, that their extension depends chiefly on the strength of the irritant and on the degree of irritability; that in the reflex-functions the posterior roots of the spinal nerves serve exclusively as exciting, the anterior exclusively as reflecting nerves; that the activity of the irritants which produce reflex-motions, is modified and increased by the peripheric expansion of the nerves; that irritation of the sympathetic nerves of frogs excites widely extended reflex-motions; that the conducting of the nervous principle from the periphery to the central organs and from there backwards to the peripheric nervous expansions, is not subject to the same laws as the conducting process in the nerves, it not being confined to the course of the nerves. According to Volkmann the present state of our knowledge is not sufficient to prove that all the reflex-motions of decapitated animals, and especially of decapitated amphibia, go on without the co-operation of mind, as the principle of sensation and of the will.

Carus is the next physiologist whose opinions on the reflex-function are worth considering. With respect to the term "reflexion," he says that as a term it is unobjectionable, only that we should beware of introducing into nervous life the idea of some new distinct agent. According to Carus the spinal marrow contains not merely primitive fibres, but also a pulpy mass, and in so far peculiar feelings and re-actions appertain to it just as much, as the capability of conducting; nay, by this mass the oscillation of the primitive fibres passing through it must undergo a modification every time in a certain proportion. Hereby also it may be understood further why, when the oscillation of the innervation in the spinal marrow to the brain is interrupted by any cause, as, for instance, directly by dividing it, nevertheless nervous feeling and re-action must still proceed from the spinal marrow itself. Carus rejects the division of the nerves into spontano-motory and reflecto-motory; he further says that, by M. Hall's theory, physiology has been encumbered with a number of superfluous names; he asserts that the admission of reflexion, as a distinct power, is altogether needless. According to him nothing whatever occurs in this case in the central nervous system, but what occurs repeatedly and almost universally in the sympathetic nervous system, viz. that the sensible nervous oscillation, instead of proceeding along from the peripheric bending of the primitive fibres to the central bending, meets the nervous mass on its way, on which feeling is concentrated and immediately springs round to re-action, so that it extends itself from here through other radiating primary fibres back to the periphery, and gives rise to convulsive twitches, &c. &c. Several other distinguished physiologists have expressed themselves in very decided terms on the merits of the reflex theory. To detail their opinions on this subject would be altogether out of place in our Journal.

With respect to the early use of the term "Reflex," we know that Haller has frequently used it in his Physiology. Unzer, who published a work on physiology in 1771, uses the term many times. His definition of reflexion is, a change of an external into an internal sensible impression. Treviranus also uses the term in his Biology or Philosophy of Living Nature, published at Göttingen, 1818. Fred. Arnold, in his work entitled Kopf-theil des Vegetativen Nervensystems, published in 1830, makes frequent use of the term reflexion; thus his experiments induced him to admit that the action of light does not take place immediately on the retina, but that it is *reflected* through the nervous expansion in the eye to the iris, and that this *bringing back* of the stimulus of light takes place through the brain. He also distinguishes the motions of the tympanum, which arise in consequence of an irritation *reflected* from the auditory nerve to the moving apparatus of this nerve, from those which immediately follow through the vibrations of the air. The sympathy of the thoracic and abdominal organs with the brain and organs of sense he also accounts for on the principle of reflexion.

When we come to consider *the Facts on which the Reflex-theory is founded,* we shall find that they possess still less of novelty than the theory itself. It is long known that the life of certain animals may sometimes continue for some time without a cerebrum and cerebellum, and that amphibious animals, after taking off their head, are still capable of making suitable movements; they seek to avoid injuries, and fly from dangers. Several facts appertaining to this point are to be found in Haller, Kaau, Boyle, &c. We shall here cite a few. Robert Whytt, proving that the nerves are the sole organs of sensation, says: if, immediately after decapitating a frog, one of the toes of the hind-foot be wounded, either very slight motion, or none at all takes place in the muscles of the foot. But if we pinch or wound this animal's toe ten or fifteen minutes after the head has been cut off, then, not only the muscles of the leg and thigh, but those of the entire body, are thrown into strong convulsion, and the frog sometimes springs up violently. In this case is not the irritation of the thigh, immediately after the head is cut off, ineffectual in producing any motion in the muscles of the thigh and foot, on account of the great pain occasioned by removing the head? Whereas the muscles are thrown into motion by wounding the toe fifteen minutes after removing the head, because the pain is now so much diminished, that it no longer prevents the animal from being sensible of the pain of his wounded toe.

Gilbert Blane also has quoted some facts which are very decisive on this point. Marshall Hall has quoted them in his work on the Nervous System. Their object is to shew, that instinctive actions, even in animals possessing a brain and nerves, do not depend on sensation; that is, that instinctive or automatic acts may be performed without the intervention of the sensorium commune, and therefore without sensation or consciousness. Treviranus was induced, by experiments such as these, to deny the assumption that the faculty of association is merely a property of the brain. Legallois states it as a well-known and proved fact, that birds, whose heads were cut off, continued to live and even to run about for some time after. Mayer also ascertained the fact that decapitated animals are still capable of performing determinate and suitable acts under the influence of irritants. Thus it is evident that the facts by which M. Hall supports his theory were well known long before his time, and were employed to throw light on the doctrine of the nervous system.

### THE REFLEX-THEORY OF THE PHYSIOLOGISTS BEFORE M. HALL.

From what we have just seen regarding the use of the term "Reflex," and the early knowledge of the facts on which the reflex-theory rests, it is abundantly evident that the theory itself is not altogether new. Several authors might be adduced here, who at a very early period expressed themselves more or less in the sense of the reflex physiologists of modern times, and it might thence be shewn that the facts on which this theory is now made to rest, were from a very early period explained in the natural way.

Haller distinguishes from the motion occasioned by muscular irritability those motions which take place when the head or brain has been removed or destroyed, and which continue as long as the mere spinal marrow or medulla oblongata remain. We have already seen that Sir Gilbert Blane was cognizant not only of the facts on which this theory rests, in the acknowledgment of M. Hall himself, but that he has expressly stated that instinctive or automatic motions may be performed without the intervention of the sensorium commune, and therefore without sensation or consciousness. Robert Whytt distinguished a feeling and a rational principle of the soul, and accounted for the phenomena which take place in decapitated animals in this manner: he said that the soul, not by rational motives, as in the case of the action of the brain, but by its feeling principle, perceives external irritants, and then acts on the organs. The various sympathetic phenomena, which are occasioned by irritants, are the consequence of particular sensations, which are evoked in certain organs, and thence transferred either to the brain or to the spinal marrow, in which organs he generally seeks the true origin of all sympathetic motions. We have already had occasion to refer to the experiments of Legallois, who has concluded, from his observations on decapitated animals, that the principle of sensation and motion in the trunk and extremities has its seat in the spinal marrow, and that the life of every individual part of the trunk in particular, depends on the part of the spinal marrow from which it receives its nerves. Burdach* also, in his work on the Structure and Life of the Brain, constantly explains many of the vital phenomena in a similar manner.

We have already alluded to the distinction of the various species of muscular motion according to Hall and Müller. To enquire into the adequacy and correctness of this distinction would lead us too far from our subject;—one circumstance however in the division we cannot help ani-

---

* Karl Frederick Burdach, von Baue und Leben des Gehirns.

madverting on. When M. Hall designates the involuntary as the third species of muscular action, and states that this depends on irritability, and requires the immediate action of a stimulus on the muscular fibres, one is at a loss whether to feel more astonished at the bad logic displayed in the division of the motions, or at the confusion of the common ideas and the abuse of the most ordinary terms in physiology. Under the head of involuntary motions are understood those which are not subject to the will, do not proceed from it, and are not determined by it. That these depend on irritability no physiologist will maintain, who knows that the causes producing involuntary motions are much more deeply seated in organic life, even though the term irritability be not restricted in the ordinary sense of physiologists to muscular irritability.

However unsatisfactory and indeterminate the advocates of the reflex-theory may be in their explanation and use of the term "reflexion," yet we may safely infer that, in their employment of the word, they mean to say somewhat the same thing of the nervous principle, which natural philosophers do of light: they mean by it "a throwing back of the nervous principle into the spinal marrow, without at the same time admitting a process similar to sensation in the brain, and an effort excited thereby analogous to the will." By this name, in fact, a motion is to be designated which is essentially different from the voluntary motions following the actions of external stimuli. This meaning of the term may also be inferred from the assertion that the centripetal conducting of the nervous principle in the case of reflex motion is no longer a sensation.

With respect to what goes on in the spinal marrow in the case of reflexion, the physiologists who support the theory seem to have no very clear ideas. Thus Müller asserts that the spinal marrow in the case of reflexion does not possess sensation; he says, in another place, in accordance with this, that the seat of the sensations is neither in the nerves, nor in the spinal marrow, but that these arise through the action of the fibres of the nerves and spinal marrow on the common sensorium. He entirely contradicts himself however when, in characterising the spinal marrow to be a reflector, he understands thereby the property whereby motions follow a sensation without both kinds of nerves communicating by their primary fibres.

We now come to consider *Sensation and its Seat, as also its Relation to Reflex Motion.*

M. Hall considers sensation without consciousness a contradiction. This we know to have been the opinion of old Protagoras, who considered sensation and knowledge almost one and the same thing. This opinion was clearly refuted by Plato, who not only designates the organs of the senses as the means whereby we get sensations, but well adds, that it is only the sensations suitable and belonging to it that we can obtain by each one of those senses, and that by no one of them can we attain that sensation, to which another of these senses is set apart. This fact would suffice to refute many of the views connected with the reflex-theory regarding sensation. We shall now examine more closely the different statements on the subject separately.

M. Hall assumes that the brain is the seat of the sensations; the proof he gives of it is this, that after the removal of the head the power of sensation is destroyed. The first contradiction into which M. Hall falls, in his attempt to prove the absence of sensation after removing the head is this, that he infers this absence from the non-occurrence of motions after the action of external stimuli; whilst, however, his chief endeavour is to prove that the motions which take place in decapitated animals after external stimuli, are of a peculiar character, and not at all connected with sensation. Thus, at one time, the motions of decapitated animals following on the application of external stimuli are made to serve as a proof of the possibility of motion without sensation; whilst, at another time, the absence of these motions is brought in as a proof of the loss of the sensations. We shall now consider a few of M. Hall's experiments in relation to this point. He cut the head off a serpent: it continued to move, when it was touched, according as every following motion brought a fresh part in contact with the table. He happened to be called away, and on his return he found the serpent hanging with a third part of its body over the sharp edge of the table. He considers that a more painful situation cannot well be conceived, if we admit that the creature still possessed sensation; it must therefore be certain, according to him, that sensation ceased. Volkmann well remarks in this place how extraordinary it is that M. Hall did not interpret these results in the only way they can be interpreted, namely, that the irritability of the creature was so exhausted, that the irritants applied did not call the reflex-function into action. Another experiment of the same physiologist on this same matter is just as inconclusive. Of two eels which he decapitated, moistened with water, and placed on a table, he stuck one with several long needles. Both continued equally motionless, when they were not touched; both were equally thrown into motion on the application of any stimulus. If the least sensation remained, the eel stuck with the needles must have wounded itself. In this experiment M. Hall inferred the absence of sensation from the presence of motion, whereas, in the preceding experiment, he inferred the absence of sensation from the absence of motion. Other experiments of this same physiologist are equally deficient in proving that the sensation of a cold-blooded animal is lost with the head. Besides, M. Hall is entirely inconsistent in assuming that sensation has its seat in the brain; for he says, sensation produces two kinds of motion; the first is through the will, the second is through some passion; but some pages after he designates the proper spinal marrow as the seat of the passions and emotions.

Müller places the seat of the sensations in the brain. According to him the sensation takes place only through the action of the fibres of the nerves and spinal marrow on the sensorium commune, and the fibres act only with their cerebral extremity on the sensorium. Here however we seek in vain for proof. Both Hall and Müller wish to place the seat of sensation in the brain, because they admit of the motions which take place after removal of the head, that they go on without sensation, for which reason they do not acknowledge sensation without consciousness and will, though even here they are not entirely consistent.

The conclusion which M. Hall drew from his experiments, viz. that sensation is abolished in all parts of the body situate beneath a division of the spinal cord, and that only excito-motory motions remain, is then only true, when the term sensation is restricted to perceptions attended with consciousness. Müller also says that we have no certain facts to shew that the spinal marrow possesses sensation independently of the brain and medulla oblongata. He also maintains that after the removal of the head reflex motions take place without sensation, but censures M. Hall for admitting that, in a perfect and sound state of the body, every motion following on a true sensation is occasioned by the will, and that all excitation of sensible parts in reflected motions is without sensation. This contradiction however arose from inconstancy in the use of the term sensation.

### Physiology of the Spinal Marrow in so far as it is related to the Reflex-Theory.

In order more closely to examine the Reflex-Theory, which represents a part, and indeed a very important part, of the functions of the spinal marrow, our author proposes to consider the opinions of the reflex-physiologists on the function of this part of the nervous system. These physiologists distinguish in the spinal chord two essentially different parts; the one serves for conducting the impressions from the nerves to the brain, and from the latter to the former; the other is the middle point of the so-called reflex-function.

Into the consideration of the intervertebral chord of sensitive and voluntary nerves, which go to and from the brain as their middle point, M. Hall does not enter very minutely. Müller, on the contrary, determines it to be the property of the spinal marrow to serve as a conductor of the nervous principle, or of its oscillations. According to him the spinal marrow represents, or is connected with, not only all the nerves of the trunk in general within the brain, but also the individual primary fibres of these nerves. The brain receives the impressions of all the sensitive fibres of the entire organism, becomes conscious of them, and knows the place of the sensation according to the affection of the various primary fibres and of the spinal marrow in the case of voluntary motion. He admires in this action an infinitely complicated and delicate mechanism in the arrangement of the elements. So various is this power, so very similar is the action of the brain according to this physiologist in the excitement of a certain part beneath these numberless primary fibres to the performance of a many-stringed instrument, the strings of which sound, just as the keys are touched. The mind is the performer, the primary fibres of all the nerves, which are spread out in the brain, are the strings, and the commencements of them are the keys. In another place he locates the keys of the instrument in the medulla oblongata, where they are to be acted on by the performer, the will. This view of the matter, striking as it is, and much as it may seem to bear the impress of originality, is however any thing but new, as extremely similar views are to be found in the works of the older physiologists.

Our author after disproving the claim of Müller to originality in this comparison, now proceeds to a more strict examination of the grounds on which that physiologist endeavours to establish his views regarding the structure and function of the spinal chord, so far as it is a conductor of the nervous principle. Respecting the proposition " that the spinal marrow serves as a stem or stock of the nerves of the trunk," the proof is said to be contained in the observations made after injuring the spinal marrow, according to which paralysis affects the parts which receive their nerves from this portion of the nervous system situate below the seat of injury. But this fact strictly taken proves nothing, except that the spinal marrow supplies the medium of connexion between its nerves and the brain, without warranting us on that account in calling it the stock of the nerves of the trunk. This is a hasty and arbitrary consequence from those experiments and observations, which is not borne out by close anatomical investigation, and which, our author assures us, is completely refuted by experiments to be presently detailed.

The assertion that " the spinal marrow represents, or is connected with, not only all the nerves of the trunk in the brain, but also the individual primary fibres of the nerves of the trunk," is considered to be proved by this circumstance, that " the affection of certain parts of the spinal marrow interrupts only the cerebral influence to certain muscles of the trunk, and the injuring of certain parts of the brain is followed only by paralysis of certain parts of the trunk." Did even this assertion rest on unexceptionable facts, still it would afford no proof for the above propositions, as these might be explained just as well, or even better, in another way. But unprejudiced observation and the comparison of a greater number of cases, as given by Burdach and others, shew us, that the affection of a part of the brain is no doubt frequently followed by paralysis of certain parts of the trunk, but that other and different parts may be paralysed in consequence of it, and that, on the other hand, the paralysis of a part may be the effect of changes sometimes of this, sometimes of that part of the brain. Just as little proof for the assertion in question is contained in the statement that " the unilateral cause of paralysis in the brain and spinal marrow occasions only a unilateral paralysis in the trunk." This assertion is true only with considerable exceptions. In 268 cases of paralysis in consequence of diseases on one side of the brain, 10 according to Burdach affected both sides of the body, and 258 only the one side. Among these were 15 with paralysis of the same side on which the brain was affected, and 216 with paralysis of the opposite side. In the other 27 the results were not determined. In spasmodic affections the case is different. According to the same physiologist the convulsions occurred in 25 cases on the side of the cerebral disease, in three cases on the opposite side. The power of sensation is injured in like manner sometimes on the opposite side, sometimes on the same side, where the affection of the brain exists. In wounds, which injure only a portion of the spinal marrow, but do not divide it, we find sensation and motion sometimes preserved in the parts lying beneath the injury in the human subject.

This also happens in the case of animals, in cold-blooded animals a piece of the spinal marrow may be cut out on the one side without the hinder extremities of such parts being entirely removed from the influence of the medulla oblongata and brain. The following experiment will serve as a proof of this: the head was cut off of a lively frog, the vertebral column opened in the region of the fifth vertebra, reckoned from below, and on the left side a piece of the spinal marrow was taken away about the length of the fifth vertebra, and as far as the median line. The motions occasioned by irritants continued in all the parts, but were more lively in the posterior than in the anterior extremities. The left hind-foot evinced sensibility and motion; only the motions were less determinate, and more tremulous than on the right. The two hinder extremities more especially evinced an internal sympathy: after the application of an irritant, the motions were common to both, and in perfect harmony. The community was less marked between the posterior and anterior extremities; but a difference between the right and left could not be discovered; when the left anterior extremity was irritated, slight twitchings were occasioned in the posterior extremity of the same side; but irritations of the posterior extremities did not affect the anterior in a very striking manner; their power of motion, however, was somewhat diminished. Seven minutes after applying nux vomica to the mucous membrane of the throat, tetanic rigidity set in in the anterior extremities, and the posterior were affected with twitches; but after half a minute they also became tetanic, and both with equal intensity. Tetanus was excited by irritating the left hind leg, just as by irritating any other part of the body. In general, in the rigid spasm no difference was observable from the cases in which the spinal marrow was uninjured. A similar result was obtained, when a longitudinal division of a portion of the spinal marrow was combined with the removal of a portion of it, as appears from the following experiment: the vertebral column was opened in a frog, the medulla oblongata was separated from the spinal marrow on the left side by a diagonal section, from the same part a small portion of the spinal marrow was removed, a longitudinal cut, one inch in length, was carried downwards, and thereby this part of the spinal marrow was split longitudinally in the middle. The performance of this operation occasioned some twitchings in the upper and lower extremities. Half an hour after the division, I introduced the nux vomica solution under the skin at the lower part of the back. The motions from irritation, which were quite palpable after the operation in the posterior part of the trunk, in the hind-legs and in the head, but in the fore-legs were entirely absent, increased in a striking manner ten minutes after the action of the nux vomica, and fifteen minutes after that, tetanic symptoms were established in the posterior extremities. In this case the exalted irritability as also the spasm in the right leg was manifestly stronger than in the left; still the left also was observed to be irritable, and was affected with spasms. These had the character of rigid spasm, but without being as lasting or as violent as usual. There was this peculiarity here, that the legs, more especially the right, were directed to the right, when a spasmodic attack occurred, which, however, after five minutes gradually went off. The upper extremities remained free from spasm, and from exalted irritability. When the right half of the spinal marrow was irritated in the place of the longitudinal cut, the convulsive twitches were observed only in the corresponding parts situate nearest to it; but when the place was irritated where the longitudinal incision ceased, tetanic symptoms followed in the lower extremities. Even when, on making a separation, only a small bridge remains behind, which connects both parts of the spinal marrow, the conducting power still continues, as the following experiment shews: the lower part of the spinal marrow was laid bare in a frog, after the head had been cut off. A piece of the spinal marrow was now cut out on the right side, just over the origin of the nerves that go to the lower extremities. As the animal still moved itself with considerable force, the lower part of the spinal marrow continued in connexion with the upper part on the left side only by means of a small bridge. The solution of nux vomica was now introduced with a hair-pencil into the mucous membrane of the mouth, which was immediately followed by an effort to remove the poison with the fore-feet. Eight minutes after the application of the poison tetanus was observed in the fore-limbs. If the right hind-leg was pinched, tetanus followed in the anterior part of the body, and convulsive twitches in the left hind-leg set in, but none observable in the right. Irritations of the left hind-leg were felt more intensely, they were followed also by stronger tetanic symptoms in the anterior extremities than those of the right. In reference to the conducting power of the spinal marrow, one may fairly, from these experiments, draw the conclusion that impressions affecting this organ are not conducted separately through separate fibres, but that they throw the entire spinal marrow into a peculiar state of excitation corresponding to the impression.

From the circumstance that it depends on the brain how many muscles of the trunk are moved every time, it does not necessarily follow, as Müller thinks, that the primitive fibres of the nervous trunk which go into the spinal marrow, are not bound together in the spinal marrow, but that they proceed to the brain parallel to one another, as in the trunk of a nerve.

First of all, our author contradicts the assertion that it depends on the brain how many muscles of the trunk are moved every time; for where the brain is absent, still a certain number of muscles corresponding to the occasion at the time are moved in the so-called reflex-motion. But in the normal uninjured state of the animals it does not depend on the brain, how many muscles are moved each time, at least not in the mechanical way as stated by Müller, but only so far, as that through the central organ certain motions are aimed at, which are effected through the simultaneous or successive action of a number of muscles. The will determines not the action of these or those muscles, but has reference to only this or that end, to which certain muscles must be subservient, without this reaching the consciousness of the individual so willing.

The assertion, that "if the primitive fibres of the nerves in the spinal marrow were bound together, a local sensation in the trunk would be just as little possible as an isolated contraction of separate muscles in the trunk," is just as gratuitous as it is unfounded. When Müller says that "the cause of the convulsive twitches in the brain and spinal marrow acts on separate parts in the trunk, and thus sensations are occasioned in separate parts of the trunk when certain parts of the brain and spinal marrow are injured," he probably did not consider that, in an affection of one and the same part of the brain, pains and spasms are not always occasioned in the same part of the body, and that pains, as also spasms, of a part of the body, may depend on affections sometimes of this, sometimes of that part of the brain. But even were pains and spasms of a part of the body always dependent on affections of the same cerebral part, we should be warranted in inferring from thence merely the very near relation of both parts, but not in admitting Müller's view of the matter.

Having seen that the facts, and the assertions designated as such, but so unsatisfactorily proved, in support of Müller's opinion concerning the structure and function of the spinal marrow, so far as it is a conductor of the nervous principle, are not conclusive, our author proceeds to adduce some facts which he conceives to be capable of perfectly refuting these unwarranted assertions.

1. A careful anatomical examination shews no such structure of the spinal marrow, that could support these assertions: in contradiction to them it may be stated that,

A. The spinal marrow does not diminish downwards in proportion as it gives off nerves, which should be the case, if it were the union of the primary fibres of the spinal nerves.

B. The fibres of the spinal nerves cannot be traced far into the spinal marrow.

C. The fibres of the spinal marrow, which can be demonstrated only in the white substance of the same, cannot be traced up to a certain point of the central organ; so indeterminate and contradictory are Müller's statements regarding the place where the harpsichord is to be found, whose chords are to be the primitive fibres, and whose keys the mind sets into motion.

2. Lesions in the spinal marrow contradict Müller's theory :—

A. On injuring one half of the spinal marrow we do not always observe paralysis of the parts of the same side which lie below the seat of the lesion.

B. Softening of the spinal marrow does not always destroy sensation in the parts which receive their nerves from it.

3. Experiments on animals afford decisive proof that Müller's theory concerning the conducting power of the spinal marrow is erroneous.

A. Volkman's experiments have shown that a longitudinal division of the spinal marrow does not prevent the extension of the reflex motions to all the muscles of both halves of the body, so long as any one part of the proper spinal marrow remains connected in the middle line.

B. The author has repeatedly observed the following facts in his experiments on the action of nux vomica on the spinal marrow, which he thinks peculiarly adapted to refute Muller's opinion :

*a.* The cutting out of a piece of the spinal marrow on the one side prevents not the conducting power of this part of the nervous system. The same power still continues with respect to impressions from without inwards, if the upper part of the spinal marrow is connected with the lower only by means of a small bridge, and even when an irritation of the medulla oblongata no longer readily communicates itself to the part of the spinal marrow lying below the section.

*b.* After cutting out a piece of the spinal marrow on the one side, an impression made on the sound side is more rapidly and better conducted than on the injured side; still, on the latter side also, the conducting power is quite manifest, and not very much weakened.

*c.* When, after cutting out the piece, nux vomica is applied, tetanus sometimes occurs about a moment earlier on the uninjured side. The hind-legs, also are in several cases directed to this side. This, however, is by no means constant, and never of long duration.

Having now seen that no decisive proof of Müller's theory regarding the conducting of the nervous principle in the spinal marrow can be adduced; that, on the contrary, many facts, furnished by anatomy, pathology, and experimental physiology, are undoubtedly opposed to it, we have so much the less hesitation in rejecting it, says our author, as if it be admitted, the so-called reflex motions cannot be adequately explained. In this respect A. W. Volkmann well says: if a recently-decapitated lively frog be irritated with the point of a fine needle, reflex motions take place in a great number of the muscles of the body. The irritation of the needle cannot have affected more than one or two nervous threads, and if the general motions are to be accounted for according to the known laws of nervous conduction, the irritated nervous fibre must be in immediate connexion with at least one motory fibre of every muscle. Even this connexion would not yet be sufficient. Müller very correctly says, that partial irritation of a motory nerve produces only partial irritation of the muscle, to which it is distributed, and as in the reflex motions the muscles are moved not partially but totally, the irritated fibre must be in immediate connexion not only with one motory fibre of each muscle, but with several. Now, as every fibre which conducts the irritation to the spinal marrow, is capable of producing general reflex motions, there must be many millions of nervous connexions in the spinal cord, and these numberless anastomoses must give the nervous structure the form of a capillary net-work; such a structure however is not found to exist.

After examining the views which the reflex physiologists sought to establish concerning the spinal marrow as a conductor of the nervous principle, it now remains to investigate their theory with respect to the process that goes on in this part of the nervous system in the so-called reflex motions.

For the purpose of accounting for these motions M. Hall admits a true spinal system or, as he calls it, an excito-motory system. This is to consist of a proper spinal marrow distinct from the chord of the intro-spinal nerves, and of the excito-motory nerves, which are different from the sensitive and voluntary nerves. In this system he admits the existence of a peculiar power, the excito-motory power.

As M. Hall himself has declined to demonstrate anatomically his excito-motory system, nay as he even confesses his inability to do so, the author finds it unnecessary to trouble himself with showing the impossibility of the existence of such a system by the known structure of the spinal chord. He therefore contents himself with examining the experiments of M. Hall in support of his theory. From experiments in which the so-called reflex-motions occurred in decapitated animals, and in which, after irritating the spinal marrow, motions, as well of the upper as of the lower extremities, ensued, M. Hall draws the conclusion, that the nervous action, contrary to the views of Haller and others, follows other directions, besides those of the nervous twigs and nervous fibres, directions which are incident, and reflected with respect to the spinal marrow. According to this course of the nervous power M. Hall considers that there exist certain nerves which are also incident and reflecting. These experiments of his prove nothing farther, according to our author, than that the spinal marrow and medulla oblongata have a sensibility for irritants, which act immediately on the same, that these irritants are capable of throwing this into a condition which, being transferred to nerves of motion, dispose these to excite motions; further, that the irritants which affect the sensitive nerves belonging to the spinal marrow, in like manner occasion, in this central portion of the nervous system, a state which is followed by motions. These facts, and the immediate conclusion to be drawn from them, are long known to physiologists: only the consequences which M. Hall considers himself warranted in drawing are new and also unfounded. The author is of opinion that all the experiments of this physiologist, taken together, do not contain a single fact which can fairly be deemed a proof of the existence of an excito-motory nervous system, and that the admission of such a system is not supported by any other kind of physiological proofs, and that it cannot, as M. Hall confesses, be demonstrated anatomically, and therefore that we are perfectly warranted in rejecting the theory of an excito-motory system, as a system destitute of all experimental and truly rational grounds. Having thus disposed of the substance or essence of the system, our author thinks it unnecessary to treat of the consequences deduced from it; he merely proceeds to direct attention to some errors and contradictions involved in the theory.—With respect to the distinction of the nerves on the one hand into sensitive and excitory, and on the other hand into such as serve for voluntary motion, and into reflecting, he now calls attention to some points in order to demonstrate the groundlessness and untenability of this division. When we consider the following facts, we shall feel disposed, says M. Arnold, to admit against M. Hall, that both processes, sensation and susceptibility of excitation on the one side, as well as voluntary motions and reflex-motions on the other, are effected by the same nerves:

1st. The skin, the organ from which sensations attain consciousness in so high a degree, which, as an organ of feeling, is the medium of so many sensations, is also the organ, whose irritations call forth the so-called reflex-motions, more than those of most other organs, more for instance than those of the muscles. It is accordingly here the organ through which conscious sensations are chiefly communicated, and that also through which the reflex-motions chiefly receive their excitations. The nerves, which go to the skin, are accordingly sensitive and excitory nerves.

2nd. In admitting special sensitive and excitory nerves, a general expansion of both nerves even to the most minute ramifications must be allowed to exist in the skin, every the smallest portion of which possesses sensibility and a power of excitation. For as no portion of the skin however small can be touched with the finest point of a needle, which does not excite sensation, and at the same time the so-called reflex-motions in decapitated frogs, it must be admitted, as Volkmann has well observed, that every part of the skin of the size of a needle's point contains two specifically different nervous fibres, and to every muscular fibre which is subservient to voluntary and the so-called reflex-motion, there belongs besides a cerebral fibre, also a spinal fibre.

3rd. After dividing the posterior roots of the nerves given to the posterior extremities, the animals not only lose sensibility in these parts, so that no indications of pain are observed on sticking or pinching the same, but also on irritating them none of the so-called reflex-motions follow, nor on the application of nux vomica, does tetanus take place; whilst, after touching those parts whose sensitive nervous fibres were not divided, this state immediately took place. This our author proved by experiments. He laid bare the under part of the spinal marrow in a lively frog, and then divided the posterior roots of the nerves distributed to the hinder extremities. These extremities could now be pinched and pricked without the animal evincing any sign of pain. He then applied from eight to ten drops of the solution of the extract of nux vomica by means of a hair-pencil to the mucous membrane of the mouth and the cavity of the throat. Six minutes after, tetanus came on; it was more decided in the anterior than posterior extremities; and in the latter a tetanic twitching set in, in accordance with the anterior limbs, which however was quickly followed by relaxation, whilst the tetanus held on in the parts provided with sensitive nerves, which difference was observed to exist in every new case. The hinder extremities were insensible with the exception of the inner surface of the left thigh. Irritation applied to this part was followed by tetanic spasms, whilst the hinder extremities could be irritated and pinched without tetanus being occasioned. The spinal marrow was now divided in the middle of the seventh vertebra, reckoned from below; in the parts anterior to the division the tetanus continued, but the hinder extremities were relaxed; only when the interior surface of the left thigh was touched, tetanic twitches were observed. He now divided the still uninjured sensitive nervous fibres on the left side, after which all sensation was lost in the posterior extremities, and no trace of a tetanic spasm could be observed. A repetition of this experiment was followed by the same result.

4. Nux vomica exalts so very much the power of sensation, and increases also the susceptibility to irritants after the removal of the head, that M. Hall numbers it among the poisons which produce an excess of reflex-function. This fact renders it so much the more probable, that sensation and susceptibility to excitation are not effected by different nerves and parts of the nervous system, more especially if the following points be kept in view.

A. After removing the head or brain the susceptibility to excitation still exists, and nux vomica also is still capable of producing exalted irritability and rigid spasm, a result very striking in amphibious animals. M. Arnold opened in a frog the cavity of the head as well as the upper part of the spinal marrow, and divided the medulla oblongata at its upper extremity, so that it was perfectly separated from the brain. After some minutes the vital phenomena were again re-established, and five minutes after the division the motions of irritation were plain to be seen. Now some extract of nux vomica with water was introduced under the skin on the lower part of the back. The consequence was that, after five minutes, tetanus set in a very severe degree. From this and other experiments attended with the same results, the conclusion may be drawn that removal of the brain does not stop the action of nux vomica on the nervous system, and that, after complete separation of the brain from the medulla oblongata, the action of this poison shews itself in parts which are connected with the medulla oblongata and spinal marrow, and under its influence. Accordingly these parts of the spinal marrow are capable of being thrown into a peculiar state and condition by an irritant, which possesses a specific local action on them, independently of the brain, just as after the removal of the head they produce self-dependent and determinate motions after the action of stimuli.

B. After injuring or partially removing the medulla oblongata the so-called reflex motions are weaker and of less duration, than after mere decapitation of the animals. The same thing occurs with respect to the action of nux vomica. Our author's experiments on frogs gave in general the following results in this respect.

*a.* Removal of one lateral half of the medulla oblongata does not suspend susceptibility to the action of nux vomica, if the process be carefully conducted. The action is merely of less duration.

*b.* Cutting off a small portion at the upper extremity of the medulla oblongata does not prevent the occurrence of the exalted irritability of the skin and of the tetanic spasms; it merely seems to delay their occurrence, and to be the cause of these phenomena, being less permanent in the experiments in question, and also of their being less marked in the hinder extremities.

*c.* After the division of the medulla oblongata transversely, the tetanic spasms either do not come on at all, or but very slightly and feebly.

C. After removing the medulla oblongata, the reflex motions diminish more palpably in energy and duration than after their mere lesion. This is also the case with tetanus occasioned by the application of nux vomica before cutting out the medulla oblongata.

D. The medulla oblongata through which the sensations, at least of very many parts of the body, are effected, is the great point of the nervous action of nux vomica, which increases the power of sensation in so high a degree, and also, as the reflex physiologists themselves admit, increases the susceptibility to excitation. After opening the upper part of the spinal canal in a sprightly frog, M. Arnold made, with a sharp knife, a transverse cut immediately behind the lesser brain, and a second one immediately above the origin of the nerves going to the anterior extremities. The portion of spinal marrow lying between the two cuts with the medulla oblongata was taken out and removed from the canal. In the head and in the anterior extremities all the motions of irritation were instantaneously abolished; on the contrary, they were tolerably active in the posterior limbs, and were also excited by touching the posterior part of the trunk; the hind-legs were strongly drawn up, and could not be extended without some trouble. The nux vomica was applied rather plentifully, about a grain and a half of the extract mixed with water, partly beneath the skin on the back, and partly on the outer skin. Fifteen minutes later no tetanic spasms had as yet appeared, but the motions of irritation began to decrease. After seventeen minutes the motions of irritation were only very weak, but no convulsive twiches could be perceived. In general such effects did not follow.

From these experiments, as well as from the circumstance that the tetanus occasioned by the application of the nux vomica still continues if the medulla oblongata is not removed till after the action has set in, the following conclusion may be drawn: that the spinal marrow is not active from its own intrinsic powers, and that it re-acts only so far as it is charged from the medulla oblongata, and according to the manner in which this charging has taken place. One might then consider the so-called reflex-motions still continuing after the removal of the medulla oblongata, as charges of the nervous fluid accumulated from the spinal marrow previously transferred from the medulla oblongata. This view however is but partially correct; for, besides the structure of the spinal marrow and so many physiological grounds, it is opposed by the circumstance, that certain agents still act on it after the medulla oblongata has been removed; strychnine, for instance, is still capable of exciting rigid spasm under favorable circumstances.

Müller does not express himself very definitively, regarding what takes place in the spinal marrow in the reflex-motion. According to his view an irritation of a sensorial spinal nerve next causes a centripetal action of the nervous principle to the spinal marrow. If this can reach the *sensorium commune,* a conscious sensation is produced. But if it does not reach the *sensorium commune* in consequence of a division of the spinal marrow, it still retains its entire power as a centripetal action of a sensorial nerve to produce a reflex motion. In the first case the centripetal action would be at the same time a sensation, in the latter case not, but it is sufficient for the reflex-motion or for the centrifugal reflexion. But we are not here told in what way the centripetal action, which does not reach to a conscious sensation, brings about a centrifugal reflexion, and wherein the process effecting this in the spinal marrow differs from that in the brain in the motions attended by conscious sensations. From more than one passage in Müller it appears that he assumes in the spinal marrow a mechanical transference of the nervous fluid from the nerves of sensation to those of motion. This assumption however is contradicted, not only by all the facts above adduced to disprove the isolated conducting property of the nervous fibres in the spinal chord, but also by the experiments which show that the motions of irritation of decapitated animals evince the character of determinateness and harmonious accordance.

We shall now close our notice of this work by presenting an analysis of the last chapter, entitled "Facts which the Observation of Nature presents regarding the so-called Reflex-Motions, and Consequences therefrom."

Close observation presents, regarding the so-called reflex-motions in decapitated animals, the following facts:

1. If the convulsive movements which frequently follow decapitation are over, or if the animal has recovered from the paralytic state, which sometimes sets in after it, it generally performs motions only on the application of external stimuli. In decapitated frogs no doubt self-dependent motions are sometimes observed to follow: they occur, however, when the frogs are placed in unusual positions, and consist in their endeavouring to attain their ordinary position, or one more convenient. Such movements however are only apparently self-dependent; they are essentially occasioned from without.

2. The motions in decapitated animals, occasioned by external irritants, bear the character of suitableness, and when combined, they indicate internal connexion and harmony. The end generally shews itself in the motions, such end being to remove the external irritant which gives rise to them, and frequently considerable exertion is made for the attainment of this end. If, for instance, a decapitated frog be laid hold of in the back with a pincers, he springs with one of the hind-legs at the place and seizes the pincers. Oftentimes the wish to withdraw itself from the irritant, is the palpable cause of the motions. The animal extends the irritated limb, the frog jumps from it, if the irritant be severe.

3. The so-called reflex-motions are more lively in decapitated than in undecapitated amphibia.

Whilst in undecapitated frogs it happens frequently that no motions take place after external irritants, when, for instance, the animals observe that they cannot instanter withdraw themselves from the irritant; determinate and suitable motions in general follow after cutting off the head or removing the brain in consequence of the action of an irritant on the skin, as soon as the animal has become tranquil and its irritability is again re-established.

4. The briskness of the motions is increased by those influences which suspend or oppress the activity of the brain. If opium be given to an undecapitated frog, our author has found that, in addition to the state of stupor, infringement on the will and on the free selection of its movements, there is observed an exalted sensibility to external irritants in the skin, and hence a greater liveliness in the so-called reflex-motions; there follow even tetanic symptoms, just as after the action of nux vomica. In decapitated amphibia, or in those where brain has been removed, opium no longer increases the irritability of the skin in equally strong doses, nor the motions consequent on irritations.

5. These influences, which possess a specific stimulating effect on the medulla oblongata and spinal marrow, exalt the sensibility in uninjured animals, and just in the same way heighten in decapitated animals the susceptibility to irritants, especially in the skin, and the motions resulting from irritation or the so-called reflex-motions.

6. The motions which take place in decapitated animals, depend for their character chiefly on the state of the spinal marrow induced before the decapitation, by the brain or medulla oblongata. In some degree, also, the state of the spinal marrow after the removal of the brain and medulla oblongata may be further determined by the strength and peculiarity of the external irritant, and thereby the peculiarity, strength, sprightliness, and duration of the motions may be modified. Of some animals, as birds, for instance the ostrich, it is long known that they will run after their object, even though their head be cut off while running. Whilst nux vomica, as the extract with water, does not heighten the irritability of the skin and the sprightliness of the motions after the action of irritants, and likewise occasion no tetanic spasms, if the medulla oblongata has been previously separated from the spinal marrow; so, on the contrary, the exalted irritability and sprightliness of the motions, as well as the tetanic spasms continue, if the medulla oblongata be cut out, after the effects of the nux vomica have already commenced. The circumstance that strychnine may produce tetanus even after the removal of the medulla oblongata, is an argument in favour of the self-dependence of the spinal chord.

7. The so-called reflex-motions do not cease to be general, though the spinal marrow on the one side be cut through even to the middle, or though a piece of it be cut out in the one-half. In like manner a longitudinal division of the spinal marrow prevents not the extension of these motions over all the muscles of both halves of the body, so long as one part of the spinal marrow remains connected at the median line.

8. Cutting through the posterior roots of the spinal marrow stops the so-called reflex-motions. If this division be made at the posterior extremities, and then the spinal marrow be cut transversely in the middle, the posterior extremities are perfectly motionless, which is not the case if the nerves of sensation continue uninjured.

9. On the degree of susceptibility to irritation and on the strength of the irritant depends the strength and extension of the motions from irritation, or of the so-called reflex-motions, with which also the facts 5 and 8 agree. But when the susceptibility to irritants is considerably increased, the motions lose the character of suitableness, and take on that of spasm.

10. The so-called reflex-motions take place chiefly after irritations of the skin, conjunctiva, mucous membrane of the nose, mouth and throat. Irritations of the thoracic or abdominal viscera produce no determinate, suitable movements. Even after irritations of the muscles and nerves only spasmodic twitches take place, without any character of suitableness or harmonious accordance.

We are now perfectly warranted in drawing from these facts the following conclusions:—

1. A power to feel external irritants has its seat in the spinal marrow, in some degree independently of the brain and medulla oblongata, *the perceptive faculty of the spinal marrow.*

2. This power in the spinal marrow, regards not merely the irritant in general, but also the kind, the degree, and the locality of it. But the property of perceptions connected with consciousness is wanting.

3. Next to the perceptive faculty (the faculty of feeling) is the faculty of the spinal marrow to re-act correspondingly to excitements occasioned by impressions, and in consequence of this to perform suitable, combined and harmonious motions, *the re-active faculty of the spinal marrow.*

4. These motions are no doubt suitable and harmonious. Still they want the character of freedom. They are not external manifestations of a will.

5. The spinal marrow possesses only in a slight degree the faculty of accomplishing spontaneous motions. If in decapitated animals self-dependent motions follow, they are principally the consequence of a disposition or excitation which the spinal marrow has received from the brain or medulla oblongata previous to the decapitation.

6. The degree of the perceptive faculty of the spinal marrow depends on a peculiar disposition of this organ, which is effected in it principally by the medulla oblongata, and which, without it, can be produced in this organ only in a very slight degree. The same may be said of the rapidity and violence of the motions occasioned by external irritants.

7. The disposition produced in the spinal marrow in this way continues for some time in it, if it be separated from the brain and medulla oblongata, and even in separate parts of the same.

8. That which takes place in the spinal marrow during the perception of external influences, and the determination of motions following thereon, is analogous to that which takes place in the brain during conscious sensations and voluntary motions, only that clear consciousness and freedom of the will are wanting to it, whilst the character of suitableness and of harmonious accordance appertains to it in the highest degree.

We cannot help remarking here, that this account of what takes place in the spinal marrow during its perception of external influences is very unsatisfactory, perhaps however necessarily and unavoidably so ; to attempt to explain and illustrate one thing by comparing it to something else which is equally in want of illustration, leaves us in our former condition of ignorance.

9. The impressions which the central organs receive through the nerves, produce in them a condition or disposition corresponding to their quality, depending both on the nature of the impression and on the nerve by which it is taken up and conducted to the central organs of the nervous system, whereupon these organs re-act in a corresponding manner.

10. A mere transference of the nervous principle from sensitive to motory fibres does not take place in the spinal marrow. The term " reflex-function" does not indicate what takes place in this organ during the motions occasioned by external irritants.

11. With regard to the conducting faculty of the spinal marrow, observation goes to show, that it is in its total character, in its character as a *whole*, that it imparts the condition, in which it is placed on the one hand by the brain and the medulla oblongata, and on the other hand by the nerves. After what has been already said it can no longer be admitted that the fibres of the spinal marrow conduct impressions separately and isolatedly, just like the nervous fibres.

12. It is not the number of the muscles moved that the central organ determines, but the end which is to be attained. A harpsichord-theory, as it has been introduced of late years, has no facts for and many against it.

We shall here conclude our analysis of this very interesting, and at the same time, very caustic critique of Dr. Arnold. Its perusal has more than ever convinced us that the many squabbles in science and literature, and the great multiplicity of controversies and disputes which distract the learned world, are owing less to differences of opinion regarding things, than to the abuse of words.

Much has been said to prove the antiquity of the theory of the reflex-function, and thereby to deny M. Hall's claim to the merit of originality, as its discoverer. In justice, however, we are bound to say, that even though this distinguished physiologist may not succeed in establishing his claim to absolute priority of discovery, he has the unquestionable merit of having evoked it from the tomb where it had so long slumbered, of having invested it with what it never before possessed, a determinate form and an appreciable character, and of having realised that which should be the ultimate end and aim of all physiological researches, namely, the having rendered it practically available in the diagnosis and treatment of disease.

